# *μ*-Raman Determination of Essential Oils’ Constituents from Distillates and Leaf Glands of *Origanum* Plants

**DOI:** 10.3390/molecules28031221

**Published:** 2023-01-26

**Authors:** Elli Kampasakali, Alexandros Nakas, Dimitrios Mertzanidis, Stella Kokkini, Andreana N. Assimopoulou, Dimitrios Christofilos

**Affiliations:** 1 School of Chemical Engineering & Physics Laboratory, Faculty of Engineering, Aristotle University of Thessaloniki, 54124 Thessaloniki, Greece; 2 Laboratory of Organic Chemistry, School of Chemical Engineering, Aristotle University of Thessaloniki, 54124 Thessaloniki, Greece; 3 Laboratory of Systematic Botany and Phytogeography, School of Biology, Aristotle University of Thessaloniki, 54124 Thessaloniki, Greece; 4 Natural Products Research Centre of Excellence (NatPro-AUTh), Center for Interdisciplinary Research and Innovation-Aristotle University of Thessaloniki, 57001 Thessaloniki, Greece

**Keywords:** Raman spectroscopy, volatile liquids, essential oils, *Origanum*, quantification, headspace GC-MS, in situ analysis

## Abstract

A novel, inexpensive and simple experimental setup for collecting *μ*-Raman spectra of volatile liquids in very small quantities was developed. It takes advantage of capillary forces to detain minute volatile liquid volumes. Spectra of volatile and even scattering or absorbing media can be measured more effectively. The method is used to facilitate the collection of intensity-consistent Raman spectra from a series of reference compounds present in *Origanum* essential oils, in order to quantify their constituents by multiple linear regression. Wild grown *Origanum* plants, collected from five different regions in Greece and taxonomically identified as *O. onites*, *O. vulgare* subsp*. hirtum* and *O. vulgare* subsp. *vulgare*, were appropriately distilled to acquire their essential oils. Comparison of the Raman results with those from headspace gas chromatography–mass spectrometry (HS GC-MS) confirmed the successful relative quantification of the most abundant essential oil constituents, highlighting the similarities and differences of the three *Origanum* taxa examined. Finally, it is demonstrated that directly measuring the leaf peltate glandular hairs yields exploitable results to identify the main components of the essential oil they contain, underlining the potential of in situ (field or industry) measurements utilizing microscope-equipped portable Raman spectrometers.

## 1. Introduction

Essential oils (EOs) are extracted from aromatic plants and consist of volatile compounds of various chemical scaffolds and particularly of terpenes and phenolic compounds. Depending on their composition and due to their chemical properties, EOs possess numerous biological properties, such as antimicrobial, anti-inflammatory, antioxidant and anticancer activity [1]. These properties, combined with the biodegradability and the low environmental impact of EOs, have rendered them key substances in numerous applications in the last decades. They are used in the pharmaceutical industry, in medical applications, in cosmetics and aromatherapy and as natural alternatives to biocides for domestic use or cultural heritage preservation treatments [2,3]. The extensive applications that EOs have and the high price of some of them have, unfortunately, also resulted in adulterations to increase the commercial profit. Therefore, there is a constant need for straightforward and reliable methods for the determination of EOs.

Chemical analysis of EOs for their characterization and authentication has been routinely carried out by chromatography techniques [4,5]. In GC-MS, the retention time and the mass spectra differentiate the volatile constituents, permitting the compositional analysis of the EO. The technique is of high sensitivity and can detect even tiny amounts of a compound. However, the relatively long time for sample preparation, the costly equipment and the identification of only volatile compounds constitute the main disadvantages and the reason that botanists and the industry sector explore the effectiveness of other analytical techniques for the chemical characterization of EOs, the detection of adulterants and the in situ characterization of the raw materials. 

Raman spectroscopy can provide chemical and structural information about substances through their interaction with monochromatic irradiation and the spectral analysis of the inelastically scattered light. The energy gain or loss of the inelastically scattered photons is characteristic of the elementary excitations in matter, vibrational in most of the cases, interrelated with its chemical composition and structure [6], rendering Raman spectroscopy a valuable tool for the characterization and study of matter. Further to its use in the fields of physics, chemistry and material science, the technique is routinely applied in the compositional study of materials in various other scientific fields, such as geology and atmospheric sciences, environmental sciences, forensics and cultural heritage science [7]. It is a non-destructive technique, requires minimal or no sample preparation and it does not require the use of any chemical reagents, thus lowering the cost, the environmental impact and time of the analysis procedure. These advantages also make it attractive as an analytical technique for the characterization of essentials oils extracted from aromatic plants, and such studies have been reported for the identification of their main constituents and the detection of adulterations [8,9,10,11,12]. Raman spectra of EOs were found to permit their discrimination rather easily compared to the respective ones of vegetable oils [8], whereas chemometrics analysis can be fruitfully employed when the spectra related to different plant varieties are very similar, as, for example, in the case of lavender and lavandin [13].

However, it is often the case that only small volumes of the samples and pure reference compounds are available. This, combined with the volatile nature of EOs, renders the analysis more demanding and raises questions about the accuracy and reproducibility of the Raman measurements regarding quantification. Methods for tackling the issue of volatility in Raman analysis of liquids have been reported in the literature. The use of acetonitrile as an internal analytical standard for the correction of the volatile analyte loss during measurements contributed to a short analysis time with high sensitivity for the quantification of ethanol and methanol in alcoholic beverages [14]. The experimentation with different kinds of sample carriers encountered in the literature also outlines the research efforts to minimize the evaporation rate of the volatile analytes. In a study of ethanol determination in urine samples [15], a highly reflective gold layer carrier, a cuvette with a lid and a gold coated slide with a cavity and covered with cling film were tested, in order to find the appropriate carrier regarding the minimum required sample volume and the prevention of evaporation. Similarly, different fluid sample carriers were screened for Raman measurements of human serum, initially tested with ethanol, including glass slides with a hole to trap the liquid under a coverslip, coverslip-covered O-rings on glass or quartz slides and the eventually proposed, mirror-finished stainless-steel plate with holes [16]. In the existing literature related to the analysis of EOs with Raman spectroscopy, the usage of various types of sample carriers has been reported, such as aluminum substrates [17], metal rings [10,18], quartz cells [13] and NMR glass tubes [19].

Raman spectroscopy belongs to the techniques that can be used for in situ measurements by the use of handheld modern devices of relatively high resolution, nowadays available in the market. The feature of portability has made Raman spectroscopy appealing for industries, e.g., cosmetics and pharmaceutical, mineralogy, art and archaeology, as well as for testing materials in the field [20,21,22]. Portable Raman instrumentation is also employed in the chemotaxonomy of plants, where fast and accurate information can be collected from different parts of the plant in its natural environment [23,24]. The inherent disadvantage of a relatively low signal in Raman spectroscopy, related to the low Raman scattering cross-section of substances, turns into an advantage when it comes to water, rendering this spectroscopic technique suitable for the analysis of plant material such as leaves, where moisture is present [25]. 

In the present study, the constituents of the EOs from three *Origanum* taxa were determined by Raman spectroscopy. *Origanum* was selected as a case study since, besides its biological properties and its extended applications in the food and pharmaceutical industries, it represents one of the most abundant native plants in Greece and the Mediterranean region in general. For the analysis of distillates, a novel experimental approach was introduced that is based on the use of capillary tubes and takes advantage of the capillary force to detain very small liquid volumes. The setup proved to be successful in the analysis of volatile liquids in very small quantities, by both keeping the analyte’s volume minimum and avoiding volatility issues. Multiple linear regression (MLR) was applied for the quantification of the constituents from their Raman spectra, using the measured spectra of pure compounds as reference. This approach is an alternative to the construction of a calibration curve that is frequently used in the literature [19,26,27], which requires the preparation of standard solutions of known concentrations for each compound to be determined, and thus, requires a larger amount of reference compounds. It is also an alternative to the use of partial least squares (PLS) regression analysis, where the reference set is usually provided by available sample spectra that have been already quantified by another, usually chromatographic, technique [13,18]. On the other hand, the use of MLR requires that all the spectra of the pure substances are reliably acquired strictly under the same experimental conditions, which is fulfilled by the proposed experimental setup, whereas the used minute quantities of the reference substances can be maintained, if required. The analysis of the distillates presented in this study was previously conducted with headspace gas chromatography–mass spectrometry (HS GC-MS) and the results of the two analytical techniques were compared in order to evaluate the adequacy and reliability of Raman spectroscopy for the detection of characteristic substances in EOs and the chemotaxonomy of *Origanum* members. Moreover, *μ*-Raman spectra were successfully collected directly from the leaves, and more specifically from the leaf peltate glandular hair. They exhibit very similar spectral features with the respective spectra of the isolated EOs, emphasizing the prospect of portable microscope-equipped Raman instruments for in situ characterization of the raw materials.

## 2. Results and Discussion

### 2.1. Raman Excitation/Collection Geometry

**Figure 1:**
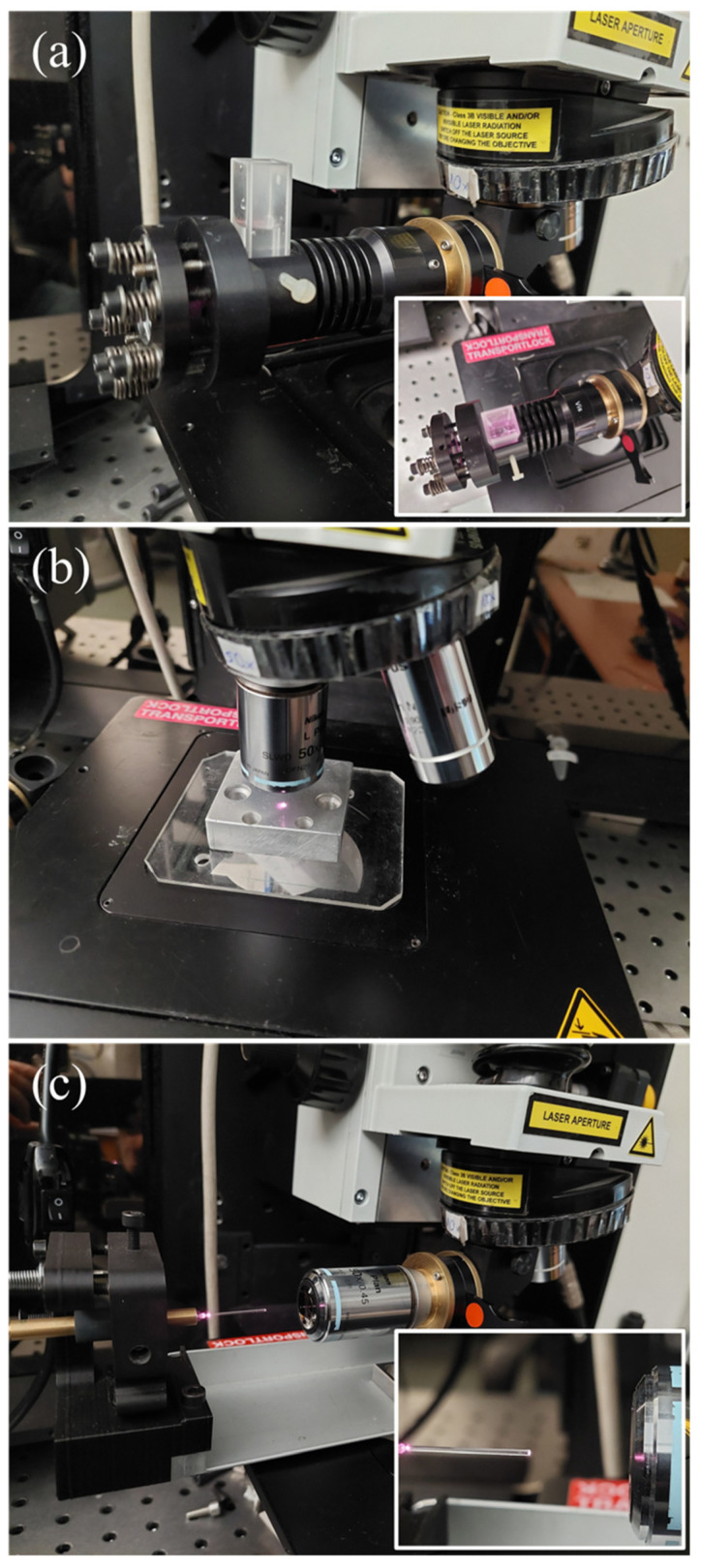
Different backscattering geometries for the excitation/collection of Raman spectra of liquids in *μ*-Raman instruments: (**a**) accessory mounted in place of an objective, permitting the use of typical quartz cuvettes, (**b**) proposed simple arrangement of a container made by drilling a blind hole in a metallic block, (**c**) proposed geometry based on the use of a capillary tube to avoid evaporation.

In much older instrumentation, a simple achromat lens of large diameter was usually employed for the collection and coupling of the scattered light to slit monochromators or spectrometers. For transparent liquids, a 90° excitation–collection scheme was common, where the light “filament”, the focused laser beam path inside the cuvette, was imaged on the horizontal slit of the spectrometer, permitting efficient coupling of the collected light. The advent of microscope-equipped Raman spectrometers, with significant benefits for the collected signal and the spatial resolution on solid samples, rendered “macro” geometries optional components, which increases the cost of the instrument. A low-cost, easy to use optional accessory that permits the use of cuvettes in the backscattering geometry of microscope-equipped Raman spectrometers is depicted in Figure 1a, and this was the first geometry to test. It is mounted in place of an objective in the microscope turret and accepts typical quartz cuvettes with an optical path of 10 mm, covered with a lid, in our case, to reduce the evaporation of the volatile components. A mirror at 45° horizontally diverts the laser beam that is focused through a simple achromatic lens into the quartz cell. A spherical mirror behind the cuvette allows a second passage of the beam, as well as collection of the forward-scattered Raman signal, practically doubling the Raman signal in our setup. The obvious disadvantage of this scheme is the requirement of large volumes. A remedy is the use of a quartz cell of small optical path, but it results in significant signal decrease and requires stringent positioning of the cuvette on the focal point, and, even so, a parasitic signal from the glass, due to the long focal depth of the lens, cannot be avoided.

Another, straightforward approach involves the use of an aluminum block with a small blind hole, filled with the liquid of interest and placed on the xy microscope table, as illustrated in Figure 1b. This arrangement allows direct excitation/collection from the free surface of the liquid, while the microscope objective of high numerical aperture ensures efficient light collection. This is an excellent approach for non-volatile liquids. For liquids of low volatility, the hole may be of small diameter (1–1.5 mm) and half-filled, reducing the evaporation by reduction of the free liquid–air interface and minimizing the required volume of the analyte. Care should be taken that the liquid level is not too low, so that the collecting angle of the objective is fully exploited. Even so, depending on the volatility of the analyte, the liquid level varies during the measurement, affecting the intensity of the spectrum. An airtight covering of the hole with a glass coverslip can significantly decrease the evaporation rate. However, for volatile liquids, condensation occurs on the coverslip, again affecting the spectrum intensity. Creating a liquid–glass interface by fully filling the hole and placing a glass coverslip over, besides requiring more analyte volume, is more prone to the collection of a parasitic signal from the glass. The use of a coverslip made from a material with a non-interfering Raman spectrum (in intensity and spectral extent), such as Raman grade CaF_2_, can mitigate the problem. In all, this approach is excellent for the collection of spectra for liquids, as long as the intensities of different analytes are not to be directly compared, because small intensity variations were observed for the volatile liquids in this study. In fact, the measurements of the investigated samples were conducted under this scheme. However, for the acquisition of reference spectra, where the same experimental conditions are strictly required, this setup is usable, but not ideal.

Holes of smaller diameters can take advantage of capillary forces to reduce evaporation; however, their cleaning becomes more difficult and time consuming. Therefore, the use of disposable capillary glass tubes was investigated. The level of the liquid inside a vertical capillary appeared less stable, probably partly due to gravity action, so a horizontal geometry was selected as depicted in Figure 1c. A capillary tube (5 μL, ~450 μm diameter) can be reproducibly placed in an optical fiber choke, attached in an in-house 3D-printed adjustable (yz tilt and position) mount. Instead, a simple mirror mount (yz tilt), in conjunction with the yz microscope stage degrees of freedom (yz position) can be used to render the laser beam coaxial to the capillary tube. Correct alignment is crucial to maximize the signal collection and eliminate the parasitic signal from the glass tube. The laser spot is focused on the midpoint of the capillary tube’s diameter, on the surface of the liquid; ~40 μm inside the liquid to maximize the probed volume, in our case of transparent compounds.

This approach yielded reproducible results since the surface of the liquid is stable for hours or considerably longer because the capillary force can detain very volatile analytes and practically eliminate the evaporation of the less volatile ones. To ensure maximum intensity consistency for the spectra of the different reference compounds, all of them were measured on the same day, one after the other, with the same alignment and calibration procedures for each. Another advantage of this method is the requirement of minute sample volumes that can also be maintained for further use, while measurement directly from the free liquid’s surface increases the signal and eliminates parasitic signals from coverslips or glass containers.

Finally, the suggested setup can serve particularly well for measurements in the case of strongly absorbing or scattering media, where the collected signal may originate only from the first few microns of the liquid at its interface with air, and strong focusing, as well as efficient signal collection are imperative for the acquisition of an adequate signal. Although this was not the case in the current study, the possibility to perform reliable measurements on volatile and/or highly absorbent or turbulent analytes renders the application of this approach more universal.

### 2.2. Raman Spectra of Reference Compounds

**Figure 2:**
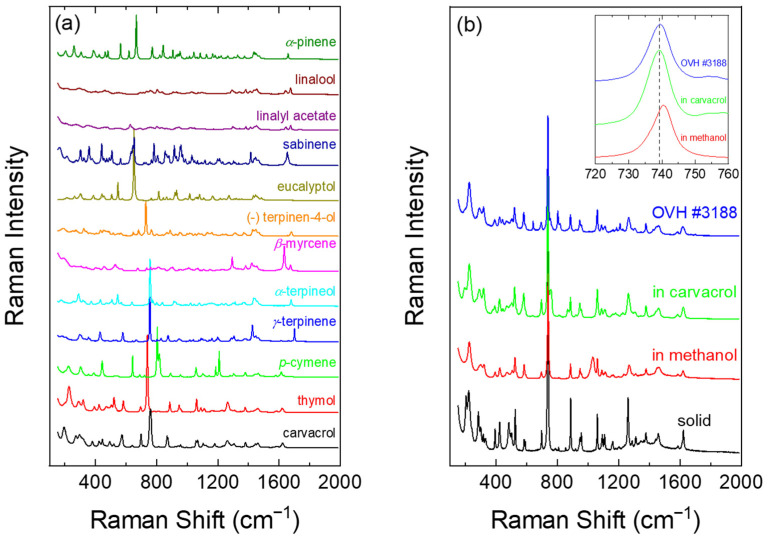
(**a**) Raman spectra acquired from available reference compounds, usually present in the *Origanum* essential oils, (**b**) comparison of the spectrum of an essential oil with high content of thymol (*Origanum vulgare* subsp. *hirtum*: OVH, #3188) with those of solid thymol, as well as solid thymol dissolved in methanol and carvacrol; the inset illustrates the spectral region of the 740 cm^−1^ thymol peak, with the vertical dashed line marking its frequency in the EO of sample #3188.

The Raman spectra of the available reference compounds were acquired as outlined in the previous paragraphs, one after the other, on the same day, and used for the qualitative and quantitative characterization of the EOs from aromatic plants of different taxa and origin. Their spectra are presented in Figure 2a.

The case of thymol was of particular analytical interest since it is in the solid state (polycrystalline) at room temperature. Comparing the crystalline thymol spectrum with the spectrum of one of the essential oils, where the thymol’s percentage was found to be high from GC-MS analysis (#3188, *Origanum vulgare* subsp. *hirtum*), differences can be observed (Figure 2b). The Raman bands of thymol in the essential oil appear broader, their relative intensities as well as their exact frequency vary, while some thymol peaks are almost absent from the essential oil’s spectrum (e.g., at ~285, 483 and 956 cm^−1^). This is not unexpected when it concerns spectra of a compound in solution and its solid phase, but it is undesirable for quantification purposes.

In order to obtain the reference spectrum of the “liquid” thymol, the solid compound was initially dissolved in methanol, which demonstrates only a few Raman peaks, none below 1000 cm^−1^, where the strongest peak of thymol (~740 cm^−1^) appears in line with [18,19]. The obtained spectrum was indeed more compatible with the one from #3188 (Figure 2b). However, the peaks’ positions differ by 0–1 cm^−1^, depending on the peak. Thymol’s strongest, and therefore of the highest statistical weight, Raman peak was shifted by +1 cm^−1^ (Figure 2b, inset). Although the value appears small, when the constituents’ bands overlap, such shifts may alter their quantification by least squares regression, favoring the more abundant compound. Eventually, carvacrol was selected as the solvent of thymol to prepare a two-component biomimetic solution, where deviations are within experimental error, by dissolving some grains of polycrystalline thymol with an adequate quantity of pure carvacrol. By measuring the intensity-consistent Raman spectra of carvacrol and the solution, the carvacrol content was deduced, its spectrum was appropriately subtracted from that of the solution and the intensity of the resulting spectrum was normalized by the thymol content to yield the reference spectrum of thymol.

### 2.3. Raman Spectroscopy of the Essential Oils from the Collected Origanum Samples

Essential oils from wild-growing aromatic plants of the genus *Origanum*, collected in summer from three localities of the Greek mainland and one in the Southeast Greek Archipelago (Table 1), were extracted by hydrodistillation, following the procedure described in the Section 3, and measured by Raman spectroscopy, as previously outlined.

**Figure 3:**
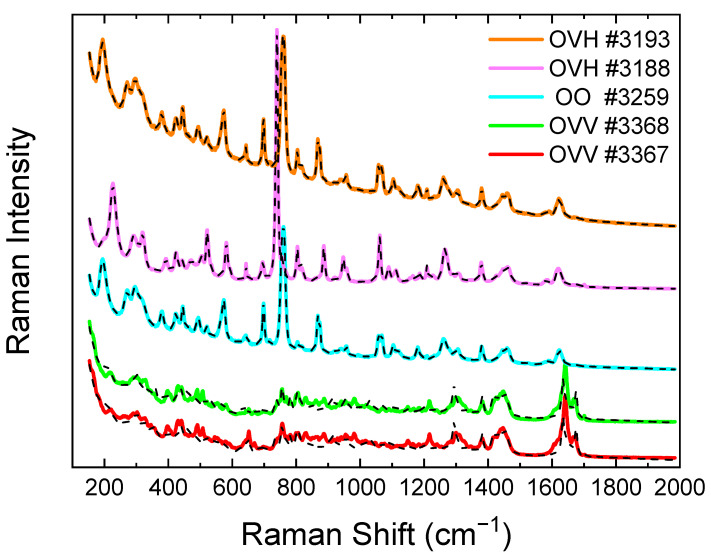
The Raman spectra of the investigated *Origanum* essential oils originating from plants identified as *Origanum vulgare* subsp*. vulgare* (OVV, #3367 and #3368), *Origanum onites* (OO, #3259) and *Origanum vulgare* subsp*. hirtum* (OVH, samples #3188 and #3193). The obtained multiple linear regression fitting curves (dashed lines) are also illustrated.

From the spectra depicted in Figure 3, the samples are easily differentiated regarding their composition, and the main constituent can be identified by comparison with the spectra of the reference compounds (Figure 2). Although both samples, #3188 and #3193, are from *Origanum vulgare* subsp*. hirtum* plants, the EO spectrum of the #3188 sample is characterized by the intense peak of thymol at ~740 cm^−1^, while in that of the #3193, the carvacrol peak at ~759 cm^−1^ is dominant, reflecting the different plant localities. On the other hand, the Raman spectrum of #3259 (*Origanum onites*) strongly resembles the one of #3193, indicating similar chemical compositions, despite their different botanical identification and the long distance between their localities. This is not a surprise since both the species of the plant and the geographical area of its origin determine the chemical composition of the essential oil, underlining the need for both chemotaxonomy and botanical taxonomy to fully characterize the taxa of Lamiaceae family [28]. The presence of *p*-cymene can also be evidenced in all spectra (e.g., doublet around 808 cm^−1^), while *γ*-terpinene in sample #3188 by a weak but characteristic peak at ~1700 cm^−1^.

On the other hand, samples #3367 and #3368 (both *Origanum vulgare* subsp*. vulgare* from neighboring localities) exhibit very similar spectra, quite different from those of the previous samples, showing no evidence of appreciable amounts of either carvacrol or thymol. The spectra seem “continuous” in the range 700–1400 cm^−1^. Based on the available reference spectra, this may reflect the presence of linalool and/or contributions of multiple compounds of similar intensity. The peaks of high intensity at around 1650 cm^−1^ may contain contributions from sabinene (~1653 cm^−1^) and *β*-myrcene (~1634 cm^−1^), while sabinene and/or eucalyptol may be reflected in the band at ~654 cm^−1^; however, no exact match can be found. The higher concentration of *γ*-terpinene in the spectrum of #3368 is evidenced by the weak but characteristic peak at ~1700 cm^−1^, as previously noted in [18,19].

These observations can be further confirmed by fitting the experimental spectra by multiple linear regression (dashed curves, Figure 3), using as a basis the available reference spectra of Figure 2a. Unlike samples #3188, #3193 and #3259, where a very good fit is observed, the fit for #3367 and #3368 is of poor quality; clear evidence that the spectra of compounds present in the EOs of these samples are not included in the spectral basis set. This is fully supported by the HS GC-MS analysis results of the same samples [28], where the available reference compounds represent only 21.5% of the identified compounds for sample #3367, while the respective value for the #3368 sample is 30.6%. *β*-Caryophyllene and D-germacrene, not included in the spectra references set, are the most abundant constituents, together representing 28.1% and 31.4% of the identified compounds in samples #3367 and #3368, respectively. Each of *δ*-cadinene, sabinene, *trans*-*β*-ocimene and *α*-farnesene exhibit values of ~5–7%, while numerous other compounds are present in percentages of 1% to 4%. Evidently, in the absence of spectra from the most abundant compounds in the samples, the contribution of the available reference Raman spectra is erroneously determined in order to fulfill the least squares requirement of the multiple linear regression, failing to reproduce the features of the samples’ spectra.

**Figure 4:**
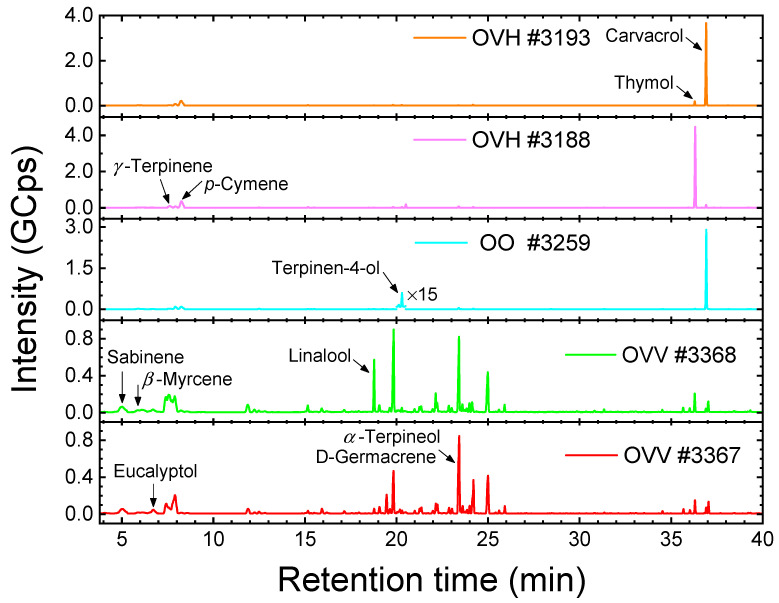
HS GC-MS chromatograms of the investigated *Origanum* essential oils, originating from plants identified as *Origanum vulgare* subsp*. vulgare* (OVV, #3367 and #3368), *Origanum onites* (OO, #3259) and *Origanum vulgare* subsp*. hirtum* (OVH, samples #3188 and #3193). Arrows indicate peaks corresponding to the available reference compounds (Table 2). *α*-Terpineol practically coelutes with D-germacrene [28]. The chromatogram of sample #3259 in the vicinity of the terpinen-4-ol peak has been appropriately magnified to enhance readability.

The Raman quantification results for each reference compound from the fitting of the #3188, #3193 and #3259 spectra are presented in Table 2, along with the corresponding ones from HS GC-MS, estimated both over the identified compounds (~98.5% of detected for all samples) and over the 12 compounds available in the Raman analysis. The chromatograms of the samples, including those of #3367 and #3368, are depicted in Figure 4.

The absolute difference in the values deduced by the two techniques (Raman, HS GC-MS) remains less than 8%. Apparently, the successful quantification relies on the fact that the reference compounds available in the Raman spectra collection constitute a percentage of 90% or higher of the identified compounds by HS GC-MS. Only in the case of sample #3188 are there other constituents estimated at 1%, namely, isopropyl-methylanisole isomers (2.7%), *α*-terpinene (1.2%) and *endo*-borneol (1.2%), while the remaining up to 100% is covered by a multitude of compounds with values less than 1%. Besides uncertainties of experimental origin, deviations may also be attributed to compounds not included in the spectral basis dataset and the probing of the liquid phase in the Raman analysis rather than the gas phase probed in HS GC-MS.

In all, the results affirm the applicability of combining Raman spectroscopy with multiple linear regression analysis for the quantification of essential oil compounds, but also underline the need for an extended intensity-consistent spectra database for successful application. Unlike the case of using PLS for quantification [13,18], which is usually based on data obtained by GC-MS, the current approach relies on data generated by the same technique (Raman spectroscopy), yielding independent results that can be compared with those from other techniques. Furthermore, calibration curves of the reference compounds [19,26,27] are not required, saving materials and time.

### 2.4. Origanum Leaves—Feasibility Investigation of In Situ Raman Spectroscopy

**Figure 5:**
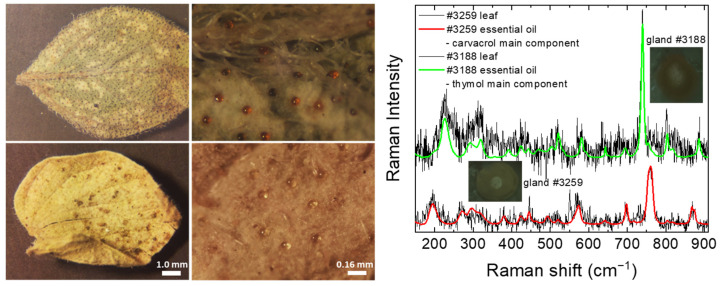
(**Left**) Photomicrographs of leaves from two taxa of the genus *Origanum*: (**top**) #3188, *O. vulgare* subsp. *hirtum* (lower leaf surface) and (**bottom**) #3259, *O. onites* (upper leaf surface). Scales are common for the images in the same column. The typical diameter of the glands is ~80 μm. (**Right**) Raman spectra (100–900 cm^−1^) from glands of the illustrated leaves, after subtraction of spectra from the edge of the glands, and their respective EOs’ spectra.

Raman spectra were also acquired directly from the leaf peltate glandular hairs of samples #3188 and #3259 (Figure 5), in order to investigate the feasibility of using *μ*-Raman spectroscopy to identify the main constituents of EOs, before any plant collection or processing is initiated, saving time, money and resources. To acquire the Raman spectrum, a filled gland is randomly selected on the leaf and the laser beam is focused a few tens of microns below its surface. Despite the very strong luminescence background in the spectra, it is possible to discriminate the strongest Raman peak of carvacrol for the #3259 (*Origanum onites*) sample and that of thymol for the #3188 (*Origanum vulgare* subsp. *hirtum*) sample.

The situation is significantly improved if a suitable reference background spectrum is recorded by focusing lower, on the edge of the gland, where the substances that contribute to the observed background are probed but not the contained EO. The resulting spectra after subtraction of this background are illustrated in Figure 5. Despite the unavoidable increase in the noise level, they compare remarkably well with the spectra of their respective EOs. Therefore, in addition to the identification of the most abundant constituent, other compounds that are present in considerable amounts may be detected, such as *p*-cymene in the #3188 sample (10.6% deduced by Raman). Its presence (3.5%) in the #3259 sample cannot be supported by the spectra, but an improvement in the signal-to-noise ratio by longer measurements may facilitate the identification of compounds in smaller concentrations. Nevertheless, the results are certainly very encouraging for the application of Raman spectroscopy in situ for the characterization of aromatic plants (native, cultivations and hydroponics).

## 3. Materials and Methods

### 3.1. Raman Instrumentation

Raman spectra were recorded using a microscope-equipped single stage spectrometer with a charge coupled detector, Peltier cooled at −70 °C (LabRAM HR, Horiba France SAS, Lille, France). Regarding the excitation laser wavelength, the typical Raman wavelength of 514 nm (Fandango 100, Cobolt AB, Solna, Sweden) was first tested. Some of the samples exhibited intense fluorescence, rendering prohibitive the general use of this wavelength. The excitation wavelength that did not create any luminescence issues on any of the samples was the one of 785 nm (near infrared) (08-NLD, Cobolt AB, Solna, Sweden). In a backscattering geometry, the laser beam was appropriately focused on the EO free surface through a Nikon 50× super long working distance (18 mm) objective, with a numerical aperture of 0.45, while the laser power on the sample was ~30 mW and the spectral resolution of the recorded spectra was ~3 cm^−1^. The typical measurement duration was 6 min. Similar was the time required for the accumulation of the Raman spectra from leaf glands but at a power of 18 mW to avoid any laser-induced effects on the glands. For the calibration of each spectrum, a Ne reference lamp was employed, ensuring an error of less than 0.5 cm^−1^. 

### 3.2. Quantification Method

The Raman signal intensity, *I_R_* = *I_R_*(*ω*)*,* is proportional to the exciting laser intensity *I_L_*, the number of available Raman scatterers—or equivalently, the probed scattering volume *V* times the number density *n* of scatterers therein—and their spectral scattering efficiency, represented by their Raman scattering cross-section *σ*(*ω*). The proportionality factor, *a*, incorporates experimental conditions such as excitation, polarization, collection and detection schemes, acquisition time, sample temperature, etc.:

(1) $${Ι}_{R}=a\;\sigma \;n\;V{Ι}_{L}$$

As long as all the experimental conditions remain the same, in the case of a liquid sample with more than one constituent, the collected Raman spectrum will be the fractional volume-weighted sum of the constituent’s Raman spectra, assuming that mixing does not result in chemical alterations or any kind of interaction between the constituents that would differentiate their Raman spectra in the mixture.

(2) $${Ι}_{R}=\underset{i}{\sum }a\;\sigma_{i}n_{i}r_{i}V{Ι}_{L}$$

Therefore, the fractional volume *r_i_* = *V_i_/V*, i.e., the *v*/*v*% concentration, of each constituent *i* can be deduced, if the individual spectra of the constituents are available. It is straight forward to use multiple linear regression, a least squares fitting with multiple variables, to fit the unknown spectrum [*y_j_*], constituted by *j* points, with the use of the constituent’s spectra [*x_j_*]*_i_*, through weighting factors *k_i_*, *k_0_* being an offset and [*e_j_*] the residuals (model’s error terms).

(3) $$\left[y_{j}\right]=k_{0}+\underset{i}{\sum }k_{i}{\left[x_{j}\right]}_{i}+\left[e_{j}\right]$$

In this approach, it is imperative that the measurement of the reference EOs’ spectra [*x_j_*] take place strictly under the same experimental conditions. Then, the coefficients *k_i_* from the fitting of the spectrum of an unknown sample directly yield the concentration of the *i*-th constituent. However, for sample analysis, it is safer to determine the relative concentration of the *j*-th constituent as $k_{j}/\underset{i}{\sum }k_{i}$, which avoids any day-to-day variation in the Raman intensity of the acquired spectra from the same experimental setup and allows analysis of spectra measured under completely different experimental conditions and different instruments. In this line, the acquisition of the references’ spectra at high spectral resolution is useful since it would permit their numerical treatment to reduce their resolution, allowing their use in the analysis of sample spectra from instruments of lower spectral resolution acquired by, e.g., portable Raman instruments.

Numerical posttreatment of acquired Raman spectra is a usual practice, which includes reasonable smoothing for noise reduction and spectral background spectrum-specific subtraction, usually polynomial. In our case, spectra were not smoothed since they exhibited a very good signal-to-noise ratio. Furthermore, no background subtraction was applied either to the references’ or the samples’ spectra. Instead, a linear and six exponentially decaying, numerically generated spectra were included in the set of the reference compounds’ spectra to account for any spectral background in the examined samples.

### 3.3. Materials

#### 3.3.1. Reference Compounds

The following compounds, usually present in *Origanum* essential oils [4], were used in analytical standard purity for the acquisition of the reference Raman spectra: carvacrol, *p*-cymene, *γ*-terpinene, *β*-myrcene, *α*-pinene, eucalyptol (1,8-cineole), (-) terpinen-4-ol and linalyl acetate, all purchased from Sigma-Aldrich, and thymol, *α*-terpineol, linalool and sabinene, all purchased from CPA Chem (Stara Zagora, Bulgaria). For the GC-MS analyses, reference compounds were diluted in LC-MS grade methanol, purchased from Sigma-Aldrich.

#### 3.3.2. Plant Material—Essential Oils’ Isolation

Aerial plant parts of three taxa of the genus *Origanum* were collected in flowering stage from three localities of the Greek mainland (Kassandra Peninsula, Mt. Pelion and Mt. Belles) and one in the Southeast Greek Archipelago (island of Kos). The aerial parts were randomly selected from ten to fifteen individuals of each locality, and geographical coordinates and altitude were recorded (Table 1). Taxonomic identification of the collected plant material was based on the key characters, as well as the descriptions given in the specific monograph of Ietswaart [29] and *Flora Europaea* [30]. Voucher specimens were deposited in the Herbarium of Thessaloniki’s Aristotle University (TAU). For the isolation of the essential oils, plants were dried in air, at ambient temperature (18–20 °C) and darkness, and leaves and inflorescences were hydrodistilled (Clevenger apparatus, 3 h). The EOs were dried with anhydrous Na_2_SO_4_ and stored in cold (4 °C) and dark conditions. For each sample, the content in essential oils (yield, in mL/100 g dry weight) is presented in Table 1.

#### 3.3.3. Headspace Gas Chromatography–Mass Spectrometry 

Headspace GC-MS analysis of the essential oils was performed as previously reported [28]. Before analysis, the essential oils were diluted in methanol at a ratio of 1:500 (*v*/*v*). Then, 20 μL were pipetted in a 20 mL autosampler headspace vial. Reference standards in methanol were also analyzed with the same chromatographic method. An EVOQ GC-TQ Bruker triple quadrupole system (Bruker Daltonics, Bremen, Germany) with a CTC-PAL autosampler (CTC Analytics AG, Zwingen, Switzerland) was utilized. The chromatographic separation was carried out on an HP-INNOWAX (30 m × 0.25 mm × 0.25 μm) column (Agilent Technologies, Santa Clara, CA, USA).

Carrier gas (helium 99.999%) was set at a constant flow rate of 1 mL/min and a split injection mode was employed. Incubation temperature and time were set at 90 °C and 15 min, respectively, agitator speed at 500 rpm and injection volume and flow rate at 1000 μL and 2 mL/min, respectively. Thermal gradient was set as follows: 52 °C for 2 min, increased to 80 °C with a 5 °C/min rate, held at 80 °C for 4 min and finally increased with a 4 °C/min rate to 250 °C for 1 min. 

Fragmentation was performed by applying Electron Impact Ionization at 70 eV. Full scan spectra were acquired from 25 to 500 amu, with a 250 ms scan time and a collection delay of 3.8 min. Injection port, ion source and MS transfer line temperatures were set at 250, 230 and 250 °C, respectively.

Data processing was performed by the MSWS 8 software (Bruker Daltonics, Bremen, Germany) and the AMDIS program (NIST, Gaithersburg, MD, USA). The NIST17 Mass Spectral Library and the retention times and mass spectra of reference compounds were utilized for the identification of the EOs’ constituents.

## 4. Conclusions

In the present study, *μ*-Raman spectroscopy was evaluated for the characterization of essential oils and the quantification of their constituents. Oregano essential oils were used as case study materials. A simple experimental setup was developed to encounter volatility and reproducibility issues during the Raman spectroscopic analysis of liquids in minute volumes. It involved the use of glass capillary tubes, which can effectively detain very small volumes of volatile analytes, saving precious raw materials. Furthermore, the proposed setup permits the acquisition of Raman spectra directly from the free surface of the liquid, enhancing the intensity of the collected signal and avoiding any parasitic signals from glass containers. The intensity-consistent Raman spectra database of reference compounds facilitated by the developed experimental protocol, combined with multiple linear regression analysis, was successfully applied in the identification and quantification of essential oil constituents of *Origanum* plants, as testified by the comparison of Raman and HS GC-MS quantifications. The latter, although time consuming, is the technique of choice since the separation of the constituents in combination with the recording of their molecular masses permit the detection, identification and quantification of all volatile compounds, even in very small quantities. On the other hand, in Raman spectroscopy, where the spectrum contains the overlapping contributions of all the constituents of the EOs, inevitably, the presence of compounds in low concentration is masked and only the most abundant ones can be detected and safely quantified. The techniques are not antagonistic since the comprehensive analysis of the EOs’ constituents that the chromatographic technique provides is counterbalanced by the simplicity and straightforwardness of Raman spectroscopy. However, in the presented approach, an extended intensity-consistent Raman spectral database of reference compounds is vital for reliable quantification.

Furthermore, the applicability of *μ*-Raman spectroscopy for identifying the main components of the essential oil directly from the oregano leaves’ glands was also demonstrated. This brings a new perspective to the characterization of plant raw material by in situ measurements, whether combining botanical and chemical taxonomy on the field, or screening raw materials for the industry.

## Figures and Tables

**Table 1:** Collection localities of wild-growing *Origanum* plants, geographical coordinates, altitude (m), essential oil content (mL/100 g dry weight) and collection code and date.

Locality	Taxon	Geographical Coordinates		Altitude (m)	Essential Oil Content	Collection Code, Date
		N (Latitude)	E (Longitude)			
Kassandra Peninsula	*O. vulgare* subsp. *hirtum* (Link) Ietsw.	40.003745	23.419388	75	5.46	#3188, 17 August 2015
Mount Pelion	*O. vulgare* subsp.*hirtum*	39.392600	22.981425	277	6.59	#3193, 10 August 2015
Island of Kos	*O. onites* L.	36.842372	27.206357	380	4.40	#3259, 28 July 2018
Mount Belles	*O. vulgare* L. subsp. *vulgare*	41.339199	23.232680	1299	0.60	#3367, 13 July 2021
Mount Belles	*O. vulgare* L. subsp. *vulgare*	41.342830	23.237160	1177	0.50	#3368, 13 July 2021

**Table 2:** Percentage fractions of the reference compounds in samples #3188, #3193 and #3259 obtained by the HS GC-MS and Raman analytical techniques. HS GC-MS results refer to the percentage of peaks’ area either over the total peaks’ area of the identified compounds or that of the available reference compounds.

	HS GC-MS % of All Identified Compounds			HS GC-MS % of the Available Raman Reference Compounds			Raman %		
Constituent	Sample								
	#3188	#3193	#3259	#3188	#3193	#3259	#3188	#3193	#3259
Carvacrol	2.2	72.2	80.7	2.4	77.1	85.9	4.4	79.7	89.0
Thymol	64.5	3.3	0.4	71.7	3.5	0.4	69.8	1.4	0.4
*p*-Cymene	16.6	14.7	9.0	18.5	15.7	9.6	12.9	10.6	3.5
*γ*-Terpinene	4.9	1.6	1.7	5.5	1.7	1.8	3.7	0.0	0.0
*β*-Myrcene	0.8	0.9	1.0	0.9	1.0	1.1	1.3	3.0	2.0
Sabinene	0.0	0.0	0.0	0.0	0.0	0.0	1.2	0.7	0.6
*α*-Terpineol	0.1	0.1	0.1	0.1	0.1	0.1	0.2	0.0	0.0
Eucalyptol	0.0	0.0	0.0	0.0	0.0	0.0	0.5	0.1	0.5
Linalool	0.1	0.2	0.0	0.1	0.2	0.0	4.2	1.9	1.6
Terpinen-4-ol	0.7	0.7	1.1	0.8	0.7	1.2	1.1	1.9	1.7
*α*-Pinene	0.0	0.0	0.0	0.0	0.0	0.0	0.7	0.9	0.6
Linalyl acetate	0.0	0.0	0.0	0.0	0.0	0.0	0.0	0.0	0.0
Sum	89.9	93.7	94.0	100.0	100.0	100.1	100.0	100.2	99.9

## Data Availability

Not applicable.
